# How Do Women and Domestic Animals Share Parturition Pain? A Systematic Review

**DOI:** 10.3390/ani16162473

**Published:** 2026-08-08

**Authors:** Ada Rota, Chiara Ottino, Alessia Bertero

**Affiliations:** Department of Veterinary Sciences, University of Turin, Largo Paolo Braccini 2, 10095 Grugliasco, TO, Italy; ada.rota@unito.it (A.R.); chiara.ottino@unito.it (C.O.)

**Keywords:** labour pain, parturition pain, physiological pain, domestic animals, woman, pain assessment, behavioural pain scales, animal welfare

## Abstract

Parturition is associated with pain in all mammals, yet our understanding of this physiological process differs considerably between women and animals. In women, parturition pain has been extensively investigated, whereas in domestic animals it remains poorly characterized, despite its potential implications for maternal welfare and neonatal outcomes. This systematic review aimed to evaluate evidence on physiological pain during natural parturition in domestic animals and to compare it with that reported in women using observer-based assessment methods. PubMed, Scopus, Embase, and Web of Science were searched from inception to 31 March 2026. Eligible studies assessed parturition pain using behavioural, physiological, or biochemical indicators. Twenty-nine studies met the inclusion criteria (18 animal, 11 human studies). In domestic animals, most investigations focused on behavioural and endocrine responses associated with dystocia or postpartum discomfort rather than on the intensity of intrapartum pain itself. The strongest evidence of severe pain during the expulsive phase was reported in goats and sows. In women, observer-based assessment methods were limited and heterogeneous. In conclusion, the available evidence is consistent with a nociceptive component of labour across species; however, it remains insufficient to quantify physiological parturition pain in most domestic animals or justify routine analgesic treatment solely on welfare grounds. The development and validation of species-specific multidimensional tools for parturition pain assessment should therefore be considered a research priority.

## Simple Summary

Parturition pain is a defining feature of childbirth. Although it has been extensively studied in women, its severity and significance remain poorly understood in domestic animals. This systematic review examines the current knowledge on physiological parturition pain in women and domestic animals, focusing on behavioural and physiological responses as well as on the methods used to recognise and measure pain. Available evidence suggests that many mechanisms and manifestations of parturition pain are shared across species, highlighting the value of a comparative approach to improve our understanding of this complex phenomenon. Nevertheless, studies specifically investigating parturition-related pain in domestic animals remain scarce. Improving the recognition, quantification and understanding of parturition pain in domestic animals could help distinguish physiological from pathological conditions, contribute to improving animal welfare and support evidence-based pain management if and when clinically indicated.

## 1. Introduction

Primitive religious beliefs suggest that suffering at parturition is a God punishment to women, but what about domestic animals? Is parturition pain similar between humans and animals? During eutocic parturition, coordinated uterine contractions and cervical dilatation are essential for the progression of labour but also generate acute nociceptive input; when labour becomes prolonged or dystocia occurs, pain and distress intensify [1,2]. It is recognized that the anatomical and neurochemical pathways of pain perception are similar between humans and domestic mammals [1,3,4] but research on parturition pain in animals remains very limited.

The second phase of parturition (labour), when active uterine contractions occur, is linked to an acute form of physiological pain, commonly rated as severe or extremely severe by most women [5]. Parturition is a complex experience, not limited to a nociceptive stimulus but permeated by emotional, cognitive, and socio-cultural factors [5,6]. Parturition pain is difficult to measure because of its multidimensional nature, in which sensory-discriminative factors, including cervical dilatation, foetal size, and primiparity, interact with cognitive-evaluative and motivational-affective components shaped by previous experiences, anxiety, expectations, and broader personal, social, and cultural influences [7,8]. These aspects contribute to the marked inter-individual variability observed in intensity and represent a challenge for pain management.

Although most mechanistic evidence derives from human studies, the anatomical and neurophysiological pathways involved in nociception are largely conserved across mammalian species [1]. This suggests that similar mechanisms underlie labour pain in domestic animals, despite obvious species-specific differences, first of all multiparity or uniparity, which warrant investigation.

Acute pain has generally been considered a normal physiological component of parturition in domestic animals [9] and, when investigated, the aim was not measuring intensity per se but to identify pain-related changes exceeding what is considered normal, which may serve as indicators of complications [1]. Quantification of pain is already difficult in animals, not only because of the absence of verbal communication, but also because pain expression varies among species, breeds, and individuals; physiological pain during parturition may be even more challenging to assess.

In women, labour pain is mostly self-assessed at different degrees of cervical dilatation and intensity is typically assessed using verbal rating scales (none, mild, moderate, and severe), numerical rating scales (NRS = 0–10, 0 = no pain and 10 = worst pain imaginable), or visual analogue scales (VAS = 0–100, a 100-mm long line ranging from 0 = no pain to 100 mm = worst pain imaginable) [10]. However, this unidimensional sensory evaluation appears inadequate to capture the multidimensional nature of parturition pain, which may require a more sophisticated multidimensional approach [11].

Behavioural observational scales have been occasionally proposed for use in women. These are based on the observation of physiological parameters and behavioural signs such as respiratory pattern, facial expression, grasping, agitation, and uncontrolled movements [6,7,12]. They offer the advantage of being less intrusive than direct questioning during the intimate and private process of childbirth. More recently, some instruments have introduced the assessment of women’s ability to cope with parturition, both physically and emotionally [13,14]. Self-assessment measures can be compared with behavioural observations [6] or clinical parameters [12,14,15] to validate observer-based parturition pain assessment tools.

Behaviour represents one of the most promising approaches for assessing pain in animals, and pain intensity can be rated using scales adapted from those developed in human medicine [16]. Largely used in non-verbal patients is the assessment of facial expressions (“grimace scales”), which is based on specific facial features associated with pain [17]. Pain in animals can also be inferred through the observation and recording of changes in physiological, neuroendocrine and biochemical parameters [16], although variations in physiological parameters (heart rate, blood pressure, pupillary dilation, etc.) or neuroendocrine mediators (cortisol, β-endorphin, catecholamines, etc.) are not specific indicators of pain and may be affected by stress, fear or disease [18].

The first aim of this systematic review was to investigate current knowledge on the severity of pain during natural parturition in different domestic animal species and to assess whether it is comparable to that experienced by women, with particular attention to its functional significance and welfare implications. The second aim was to determine whether validated observer-based scales are available for women, as a theoretical basis for studies in non-verbal patients, including animals.

## 2. Materials and Methods

### 2.1. Search Strategy

A systematic literature search was conducted in PubMed (MEDLINE), Scopus, Embase, and the Web of Science Core Collection from database inception to 31 March 2026, with no lower publication-date limit. Given the expected scarcity of studies on the topic, a broad search period was used to maximize search sensitivity. Two separate searches were performed: one for domestic animal species (cattle, sheep, goats, swine, horses, dogs, and cats) and one for women. Search terms combined controlled vocabulary (MeSH, where applicable) with free-text keywords related to parturition/labour, pain, and pain assessment (including behavioural indicators and observer-rated scales). For the human search, exclusion terms were used to reduce the retrieval of studies relying exclusively on self-reported pain measures or primarily focusing on obstetric analgesia or epidural anaesthesia; however, owing to overlap in indexing and terminology, some such records were nevertheless retrieved and were excluded during screening. Only studies that included observer-based, non-self-reported pain assessment were retained. The complete search strings for each database are provided in Appendix A and Appendix B. Searches were restricted to peer-reviewed journal articles written in English that had been formally published and indexed by 31 March 2026; preprints and articles in press were excluded.

### 2.2. Study Selection and Data Extraction

**Figure 1:**
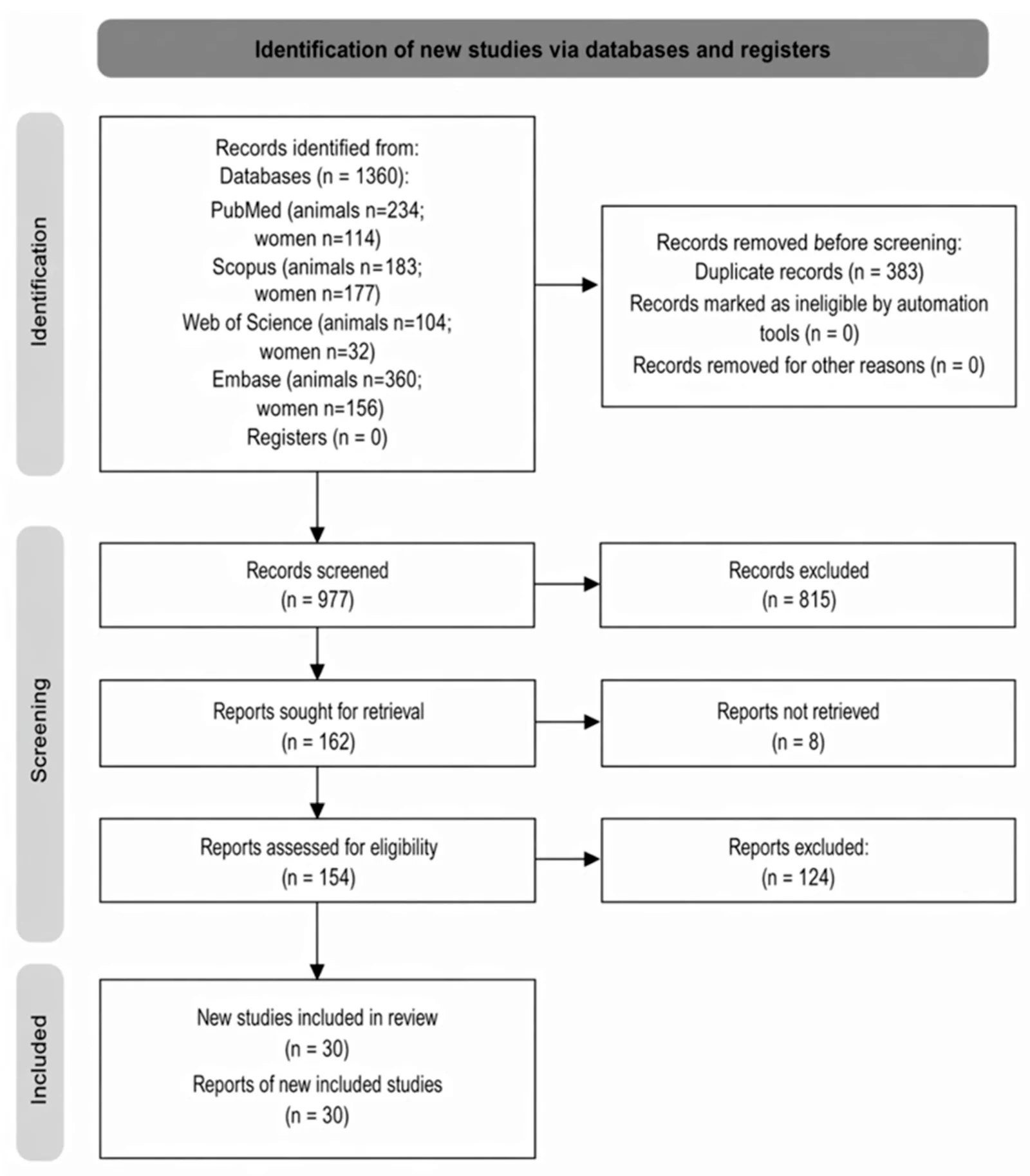
PRISMA flow diagram of the study selection.

The reporting of this systematic review followed the PRISMA 2020 Statement and its Explanation and Elaboration document [19,20]. The study-selection process is presented in the PRISMA flow diagram (Figure 1), and the completed PRISMA checklist is provided as Supplementary Material S1. Two search strategies were developed to identify studies addressing pain assessment during parturition in domestic animals and in women. Records retrieved from the database searches were exported and duplicates were removed using Rayyan (web application; Rayyan Systems Inc., Cambridge, MA, USA) [21]. Titles and abstracts were independently screened by two reviewers using Rayyan’s blinded mode to minimise mutual influence during the selection process. The full texts of potentially eligible studies were then independently assessed using the same procedure. After completion of the independent screening, any disagreements regarding study eligibility were resolved through discussion and, where necessary, consultation with a third reviewer. The same inclusion and exclusion criteria were applied throughout the study-selection process. A total of 1360 records were identified, comprising 881 records relating to domestic animals (PubMed: 234; Scopus: 183; Web of Science: 104; Embase: 360) and 479 relating to women (PubMed: 114; Scopus: 177; Web of Science: 32; Embase: 156). After duplicate removal, 977 unique records remained for title and abstract screening. Following title and abstract screening, 162 reports were considered potentially eligible and were sought for retrieval. Eight reports could not be retrieved, leaving 154 full-text articles for assessment. Of these, 124 were excluded, comprising 37 studies not published in English and 87 studies that did not address pain assessment methods during parturition. Ultimately, 29 studies met the inclusion criteria and were included in the qualitative synthesis, comprising 18 studies in animals and 11 in humans. For each included study, data were extracted on the study population (human or animal species), country of origin, study aim, and study design. Data extraction was performed using a standardized approach to ensure consistency across studies.

### 2.3. Data Synthesis

Eligible studies were organised for narrative synthesis according to three predefined descriptors: population/species, stage of parturition, and pain-related parameters measured. Animal studies were summarised by species and interpreted according to the parturition stage assessed and the types of measures reported, including behavioural, facial-expression/grimace-based, physiological, endocrine, and welfare-related indicators. Studies in women were synthesised separately using the same descriptors and were restricted to observer-based or objective assessments of parturition/labour pain; studies based on self-reported pain scales were excluded. Given the descriptive nature and methodological focus of the review, no formal study-level risk-of-bias assessment was performed. Instead, methodological characteristics relevant to the interpretation of the findings, including study design, sample size, and the presence of comparator or control groups, were extracted and considered.

## 3. Results

### 3.1. Domestic Animals

The number of retrieved studies addressing parturition pain in domestic animals was low (*n* = 18), as shown in Table 1.

The studies in cattle have primarily focused on identifying parameters useful for the early detection of complications during parturition, rather than directly assessing pain. Behavioural observations such as changes in posture, locomotor activity, lying behaviour, self-grooming, restlessness, and maternal behaviour have mainly been used to describe normal patterns and detect deviations associated with dystocia rather than to quantify pain itself [23,24,29].

In this context, some studies have integrated clinical, behavioural, and physiological approaches, including comparisons between caesarean section and natural parturition and evaluation of postpartum analgesic treatments [25,27]. However, even when physiological and endocrine parameters were considered, they were generally interpreted as indicators of systemic distress rather than as specific measures of acute pain during parturition. Hormonal fluctuations during parturition—including cortisol, catecholamines, β-endorphins, vasopressin, and oxytocin—follow the progression of labour stages and are more pronounced in prolonged or assisted deliveries in cows and goats [22]. Although these changes are consistent with a nociceptive component, they remain non-specific and do not allow clear discrimination between pain and the broader stress response. These biomarkers show distinct temporal release patterns and fulfil different physiological functions during pregnancy, labour, and the postpartum period, making their interpretation in relation to labour pain inherently complex [39]. More generally, they represent non-specific stress responses and may also increase during exercise, fasting, and other non-painful conditions. In addition, cortisol should be interpreted with caution, as it is a non-specific biomarker whose concentrations may reflect not only pain- and stress-related responses but also the physiological endocrine processes involved in the initiation and progression of parturition [40].

Further indirect evidence of pain comes from post-partum behavioural studies, in which assisted calving has been associated with alterations such as reduced self-grooming and increased time spent in lateral recumbency, both of which are frequently interpreted as indicators of discomfort. In this context, ketoprofen administration modified lying behaviour towards patterns suggestive of greater comfort. Nevertheless, these approaches rely on proxy indicators and lack pain assessment tools [26,28]. Overall, pain is rarely considered a primary outcome, and its intensity is not rated.

The effect of primiparity has occasionally been investigated in cows, although not specifically to assess pain intensity. Instead, these studies examined whether longer labour or calving difficulty affected subsequent calf vigour or the onset and quality of maternal behaviour [26], or sought to avoid misinterpreting normal behaviours in heifers as signs of dystocia [23].

In goats, the integration of behavioural, physiological, and neuroendocrine parameters demonstrated the activation of cardiovascular and endocrine systems during parturition, particularly during the expulsive phase [22,29]. As in cattle, these responses are compatible with nociceptive activation but remain non-specific. Pharmacological studies using buprenorphine did not demonstrate clear reductions in behavioural pain scores, although correlations were reported between endocrine markers, such as plasma vasopressin concentration, and pain expression [30]. More recent approaches combining behavioural indicators with facial expression analysis provide evidence of pain during parturition, particularly during the expulsive phase, with more pronounced effects in primiparous females. On a 0–10 facial grimace scale, scores obtained during the expulsive phase ranged from 5 to 9, suggesting intense pain, although the scale had originally been developed for measuring pathological pain [31].

In sows, earlier studies primarily investigated behavioural alterations associated with parturition, interpreting increased restlessness and reactivity mainly in relation to environmental constraints and individual coping ability, rather than as direct indicators of pain [32]. Composite indices such as the Ease of Farrowing Score were subsequently developed to assess parturition difficulty without specifically targeting pain [33]. More recent research has shifted towards the identification of pain-related indicators, including behaviours such as back arching, trembling, and postural changes that increase during farrowing and are temporally associated with piglet expulsion, suggesting a nociceptive origin [41]. However, even when behavioural, endocrine, and inflammatory parameters were combined, the absence of clear effects following the administration of non-steroidal anti-inflammatory drugs (NSAIDs) highlights the difficulty of detecting and quantifying pain in this species [34,36]. The development of facial expression-based tools represents a relevant methodological advancement, with Facial Action Units showing promising reliability and correlations with behavioural indicators, particularly during the expulsive phase, for which a severe-pain score was proposed [18,37]. Primiparous sows displayed a greater number of behavioural pain indicators [18], spent less time lying down than multiparous sows, and consumed fewer meals, suggesting slower recovery after farrowing [34]. However, another study suggests that pain during the immediate post-farrowing period may be greater in sows than in gilts [36]. Small sample sizes, differences in breed and parity among multiparous sows, management factors, and prostaglandin-induced parturition may account for the variability in these findings.

In multiparous species such as pigs, the expulsion of the first foetus was associated with slightly more pronounced pain-related behaviours, such as tail flick or back arch, than the expulsion of subsequent foetuses [35]. This may be explained by an endogenous β-endorphin-mediated increase in the nociceptive threshold during parturition or/and by greater mechanical distension of the birth canal, which may facilitate the passage of subsequent piglets. However, this possibility has not been specifically investigated and is not supported by other studies [37]. A very rapid and easy expulsion of the second foetus was also described in goats [22].

In horses, the available evidence is limited to a preliminary single-group observational study of 10 Polish heavy draft mares assessed during the immediate postpartum period after dystocia with fetal death treated by standing fetotomy. Because the study did not include a comparison group of mares following uncomplicated foaling, its findings reflect pain associated with severe dystocia and fetotomy and cannot be generalized to physiological parturition [38]. Notably, no studies specifically assessing parturition pain in dogs or cats were identified, highlighting a major and clinically relevant gap in the veterinary literature.

Overall, the few studies available across species support the presence of a nociceptive component during parturition, particularly during the expulsive phase, during which pain was classified as severe or yielded high scores comparable with those reported for other forms of acute pain.

### 3.2. Women

Labour pain is rated as severe or extremely severe by most women [5]. The search retrieved 108 works in which parturition pain intensity had been measured; however, in many of these studies, pain was self-assessed, usually using a visual analogue scale [42].

The review by Capper & Ferguson (2024) confirms that the visual analogue scale and the numerical rating scale are the most commonly used unidimensional tools for reporting labour pain intensity in women [11]. More recently, the Wong–Baker Faces Pain Rating Scale, originally developed for children and comprising a series of six cartoon faces, was used to communicate the intensity of labour pain [43]. Improving the assessment of parturition pain by accounting for its physiological, emotional, social, and cultural components remains a major challenge in promoting women’s care and well-being; the ‘McGill Pain Questionnaire’ is among the most widely used multidimensional self-assessment tools [11].

However, because these tools cannot be used to measure pain in animals, for which self-assessment is impossible, we focused on studies of women in which parturition pain was assessed using behavioural observations and/or clinical parameter determination. These studies are summarised in Table 2.

We identified only eleven studies that explicitly report the use of non-verbal behavioural scales in women, despite the growing emphasis on respecting the privacy and intimacy of childbirth and avoiding unnecessary questioning during labour [6]. A rating scale for labour pain based exclusively on midwives’ observations offers the advantages of minimising interference with the delicate and intimate birthing process and overcoming language barriers [6].

The study by Bonnel & Boureau (1985) proposed a 0–4 scale based on the observation of respiration, agitation, grasping reactions, and uncontrolled movements, and evaluated the relationship between behavioural scores and patients’ self-report scores, which were significantly correlated [7]. Another study showed that caregivers correctly assessed labour pain in more than half of the women [15], although there was no significant correlation between midwives’ and mothers’ ratings of pain intensity during stage III of parturition [44]. Another study aimed to identify clinical indicators of pain during labour and examine the association between the clinical nursing diagnosis, verbally reported pain intensity, and uterine contractions [12]. The observational scale developed by Bonnel & Bureau (1985) [7], was used to evaluate the effect of an immersion bath during the first clinical stage of labour on pain intensity [45], while other behavioural scales are occasionally described and were used to assess the effectiveness of massage in reducing pain and anxiety during labour [46], and of devices such as the peanut ball [47]. Scales based on labour observation were also used to assess the effects of breathing exercises and virtual reality applications on pain intensity [48].

Alternative tools have been developed to capture additional dimensions of labour pain. The Coping With Labor Algorithm was proposed as a less intrusive approach that avoids directly questioning women about pain intensity during the birthing process while also assessing psychological aspects [13]. More recently, the Coping Assessment for Laboring Moms (CALM) scale was introduced as a multidimensional tool to support nurses in assessing how women cope with labour. It evaluates coping on the basis of facial expressions, behaviour, and vocalization and may enhance women’s perception of nursing presence during labour, thereby increasing their comfort [14].

**Table 2:** Main characteristics of the retrieved studies in which parturition-related behavioural traits have been measured in women.

Ref.	Study Design	Country	Sample Size (*n*)	Parity	Observation Period/Stage	Study Aim	Assessment Method
[7]	Prospective methodological validation study	France	100	Primiparous	Labour	Development and validation of an observational tool for assessing labour pain	Behavioural pain scale (0–4)
[44]	Prospective longitudinal observational study	Sweden	138	Mixed	Labour	Evaluation of expectations regarding labour pain and agreement between maternal and midwife pain rating	3-point rating scale
[15]	Prospective comparative observational study	Beer-Sheva (Israel)	255	Mixed	Labour	Identification of factors influencing caregivers’ assessment of labour pain	Visual analogue scale (VAS)
[46]	Randomized controlled trial	Taiwan	60	Primiparous	Labour	Evaluation of the effect of massage on labour pain and anxiety	Present Behavioural Intensity (PBI) scale
[45]	Randomized controlled trial	Sao Paulo, Brazil	108	Primiparous	Labour	Evaluation of the effect of immersion bathing on labour pain	Behavioural pain scale
[13]	Prospective quality-improvement study	Salt Lake City USA	Not reported	Not reported	Labour	Implementation of the Coping With Labor Algorithm in clinical practice	Coping with Labour Algorithm
[12]	Prospective clinical validation study	Sao Paulo, Brazil	55	Primiparous	Labour	Validation of clinical indicators of labour pain	Clinical indicators of labour pain
[14]	Prospective comparative study	Massachusetts (USA)	Not reported	Not reported	Labour	Comparison of the CALM scale and the Numeric Rating Scale	Coping Assessment for Laboring Moms (CALM) scale
[6]	Prospective scale-development and validation study	Spain	55	Mixed	Labour	Development and validation of an observational scale for pain expression	Rating Scale of Pain Expression during Childbirth
[47]	Protocol for a randomized controlled trial	Karnataka (India)	550	Primiparous	Labour	Evaluation of the effectiveness of a peanut ball during labour	Behavioural response observation checklist
[48]	Single-blind randomized controlled trial	Turkey	114	Primiparous	Labour	Evaluation of the effects of breathing exercises and virtual reality during labour	Labour observation form

## 4. Discussion

Pain is a defining feature of human parturition and has long been described as one of the most intense and exhausting forms of acute pain that a person may experience, imposing considerable physical and psychological demands [5]. At the same time, parturition pain differs from pathological pain because it arises during a physiological process rather than from disease. This dual nature may explain why it is not experienced solely as aversive but may also be perceived as meaningful, purposeful, and productive because it leads to childbirth [8]. Most women fear labour pain and a primary objective in obstetrics is to alleviate it and promote maternal well-being. Accordingly, most of the studies on parturition pain in women identified in our search evaluated the effectiveness of interventions intended to relieve it, ranging from antenatal education and training to pharmacological treatments [49,50,51], alternative therapies [50,52], complementary approaches such as music and aromatherapy [53], and devices such as the peanut ball [54].

From a pathophysiological perspective, parturition pain is a complex and multidimensional phenomenon arising from the interaction of mechanical, neuroinflammatory, and neuroendocrine mechanisms [1]. Uterine contractions, cervical dilation, and foetal descent activate visceral and somatic nociceptors located in the myometrium, cervix, and pelvic structures [55]. During the first stage of parturition, pain is predominantly visceral, transmitted through unmyelinated C fibers via sympathetic pathways to the thoracolumbar spinal segments, and perceived as diffuse and poorly localized. As labour progresses, a somatic component emerges as a result of distension of the birth canal and pelvic floor, producing sharper and more localised pain [56]. Nociceptive processing may be further amplified by peripheral and central sensitization mechanisms. The local release of inflammatory mediators, such as prostaglandins and bradykinin, lowers nociceptor activation thresholds. In particular, prostaglandins sensitise peripheral nociceptors and enhance their responsiveness to the mechanical and chemical stimuli generated by uterine contractions and cervical dilatation while repeated afferent input increases the excitability of spinal neurons; both mechanisms contribute to the progressive increase in perceived pain intensity during labour [57]. Labour pain is closely linked to neuroendocrine regulation, with several interacting systems contributing to both the generation and modulation of nociceptive input. Oxytocin plays a central role in uterine contractility and may also influence pain perception, while activation of the hypothalamic–pituitary–adrenal axis leads to increased cortisol levels, reflecting the physiological stress response. At the same time, endogenous opioid systems, including β-endorphins, are activated and contribute to the modulation of nociceptive thresholds, thereby providing an intrinsic analgesic mechanism [39,58,59]. These findings support the existence of intrinsic antinociceptive mechanisms, but they do not resolve the challenge associated with measuring pain.

Physiological and neuroendocrine variables may help characterise parturition pain, but they do not provide a direct or specific measure of its intensity. Circulating catecholamines and glucocorticoids reflect autonomic and hypothalamic–pituitary–adrenal activation and may therefore be considered indirect correlates of pain and stress, although they are of limited value in quantifying pain itself [1].

Our results confirm that research interest on animals’ parturition pain has been driven more by the potential consequences of dystocia for productivity, reproductive performance, and neonatal viability than by an interest in directly assessing pain from a speculative or welfare perspective [1,4]. This difference likely reflects the historical priorities of veterinary research, particularly in livestock species, where production objectives and economic considerations have largely shaped research questions. Consequently, studies have mainly focused on identifying indicators of dystocia and compromised maternal or neonatal outcomes, whereas the assessment of physiological labour pain as a welfare issue has received comparatively little attention. Consistent with this interpretation, no studies specifically addressing physiological parturition pain were identified in companion animals.

Compared with the human literature, the evidence available in domestic animals is considerably more limited and conceptually fragmented. Although the existence of pain-related mechanisms during parturition is consistent with the conservation of nociceptive pathways across mammals [1,3,4], the intensity and biological significance of parturition pain in domestic animals remain poorly defined.

In the few studies that explicitly addressed the painful component of parturition in sows [18] or goats [31], parturition pain intensity was classified using categories such as mild, moderate, or severe. The pain scales used in these studies had previously been applied and validated in animals subjected to painful procedures or surgeries, or affected by pathological conditions [18,31,60], and combined behavioural indicators (e.g., back arching, tremors, and postural changes) with facial expression-based approaches [18,37]. A method of pain assessment should ideally be validated against a “gold standard”; however, since a “gold standard” method of animal pain assessment is lacking, pain indicators must be thoroughly assessed by comparing animals before, during, and after a painful intervention or event; by comparing animals presumed to be in pain with control animals; and by evaluating the effects of analgesic treatments [41].

Pain was rated as severe during the expulsive phase in goats and, in pigs, particularly during the delivery of the first piglet [18,31]. Other available studies on domestic animals support the presence of pain-related responses during parturition, but they do not allow firm conclusions to be drawn regarding the absolute intensity of parturition pain or its comparability with the pains experienced by women. The effects of primiparity and the order of foetal expulsion in multiparous species on parturition pain intensity require further investigation.

Human parturition pain is characterised by remarkable inter-individual variability. Its intensity, temporal progression, and spatial distribution may differ substantially among women, with pain radiating across the abdomen, flanks, and back and changing as labour progresses [5]. Self-assessment of pain has inherent limitations during parturition, because ratings are obtained at discrete time points and may be influenced by the stage of parturition, the duration of contractions, fatigue, and the woman’s ability or willingness to respond while coping with birth [7]. Moreover, neither the multidimensional experience of parturition nor the meaning and complexity of parturition pain can be adequately captured by unidimensional tools such as the Visual Analogue Scale and the Numerical Rating Scale which mainly measure the sensory-discriminative dimension of pain intensity [8,10,11]. The studies identified in women that used non-verbal or observer-based tools are particularly relevant to the aims of the present review. Behavioural observational scales based on restlessness, body movements, facial expression, vocalisation, perspiration, and coping-related behaviours have been proposed as less intrusive alternatives to direct questioning [6,7,12]. Although discrepancies between caregiver ratings and maternal self-report may occur, observer-based approaches have shown meaningful correlations with patient-reported pain in acute settings [61] such as labour and may be especially useful when repeated verbal assessments are undesirable or impractical [7,15]. More recent instruments, such as the Coping with Labor Algorithm and the Coping Assessment for Laboring Moms scale, have extended this approach by attempting to capture not only pain expression but also coping and emotional adaptation during labour [13,14].

These experiences in women provide a valid theoretical support for comparable studies in animals.

In women, parturition pain is clearly multidimensional, because sensory input is intertwined with meaning, expectation, fear, coping, and cultural and social factors [8]. Although some of these components may be absent or less developed in animals, the experience of parturition undoubtedly includes an emotional component. It is reasonable to assume that domestic animals do not experience the same culturally mediated anticipation, symbolic meaning, or cognitive appraisal that shape human childbirth [62]. However, this does not imply that parturition pain in animals is purely mechanical or negligible. Animals, like women, undergo profound physiological changes during parturition and must rapidly adapt behaviourally to the demands of labour and neonatal care. Species-specific pain expression should be interpreted in light of evolutionary ecology and domestication. Anti-predator behaviour may minimize overt signs of vulnerability. The absence of conspicuous vocal or motor responses in studies of cattle, sheep, and goats should then not be interpreted as evidence of milder pain. Social organisation, individual temperament, previous experience, habituation to humans, and selective breeding may also affect the expression of pain-related behaviour, although their specific influence on labour pain remains poorly established. Cross-species comparisons should therefore rely on convergent behavioural, facial, physiological, and endocrine indicators rather than on overt behaviour alone [62,63,64]. From this perspective, parturition pain in animals may best be regarded as a physiological form of pain that nevertheless deserves investigation within a welfare framework. This raises the more speculative question of whether parturition pain may have a functional role in facilitating the physiological and behavioural adjustments associated with parturition. One hypothesis is that parturition pain helps direct appropriate behaviour in women and other animal species by focusing attention on the demands of birth, giving a starting signal to concentrate on this task and completely dedicating to it [65]. From an evolutionary perspective, acute pain is not only a consequence of nociception but also a motivational system that prioritises protective behaviour and signals the need for assistance to conspecifics. Parturition pain has thus been hypothesised to direct the parturient towards safety and, in women, to recruit social support, with species-specific responses ranging from seeking support during birth to isolation seeking in cattle [66,67,68] and nest-building in sows [69,70]. These observations are compatible with an adaptive role but do not demonstrate it, as such behaviours also precede active labour and are hormonally regulated. Whether parturition pain has an adaptive role, and why such pain may be necessary, remain unsolved questions in both humans and animals.

Endogenous opioid mechanisms may be relevant in this regard. Increases in β-endorphin and opioid-mediated changes in nociceptive threshold have been reported around parturition in cattle and sows, suggesting the existence of intrinsic systems that may help the mother to cope with labour under physiological conditions [71]. Consistent with this interpretation, animals often appear able to cope with normal labour, whereas clearer signs of suffering and dysregulation emerge when parturition becomes prolonged, assisted, or complicated. This does not mean that physiological parturition is pain-free; rather, it indicates that current evidence is insufficient to determine whether and when physiological pain exceeds an acceptable threshold and becomes a welfare concern requiring intervention. Overall, the comparison between women and domestic animals highlights both the biological continuity of parturition pain across mammals and a major gap in knowledge. In women, parturition pain is recognised as severe, multidimensional, and difficult to measure although structured observational tools are increasingly available. In animals, by contrast, parturition pain must be inferred indirectly, and the available evidence does not permit definitive conclusions regarding its intensity or comparability with women’s experience. Current knowledge therefore does not support recommendations for the treatment of physiological parturition pain in domestic animals solely on welfare grounds.

### 4.1. Study Limitations

One limitation of this review is the restriction to English-language publications, which may have introduced language bias by excluding potentially relevant studies published in other languages. However, only a limited number of non-English articles were identified during the screening process, suggesting that the impact of this restriction on the overall findings is likely to be limited.

In addition, only peer-reviewed journal articles were considered eligible, while conference proceedings and other forms of grey literature were excluded. Although this criterion was adopted a priori to ensure the methodological quality of the included evidence, it may have increased the risk of publication bias, as preliminary studies or studies reporting negative or inconclusive findings may not have been captured. This aspect may be particularly relevant in veterinary medicine, where early findings are frequently presented at scientific meetings before publication as full-text articles.

Finally, the included studies were few and highly heterogeneous in terms of species, study design, sample size, stage of parturition, and outcome measures. This heterogeneity precluded a meaningful meta-analysis and limits the generalisability and direct comparability of the findings. In addition, many animal studies were not primarily designed to assess pain and relied on indirect behavioural, physiological, or endocrine indicators that are not specific to pain; several assessment tools had also not been validated specifically for parturition.

Evidence from human studies was deliberately restricted to observer-based assessments, whereas self-reported pain is regarded as the primary measure of pain intensity in communicative patients. Accordingly, the cross-species comparison should be interpreted primarily in terms of observable pain-related responses and assessment approaches, rather than as a direct comparison of subjective pain intensity.

An additional limit is that no formal risk-of-bias assessment of the included studies was performed. Although this review was intended primarily to map and compare pain-assessment approaches across heterogeneous study designs rather than to estimate intervention effects, this omission limits confidence in the interpretation of individual studies and in the inferences drawn from the overall literature. The findings should therefore be regarded as descriptive and hypothesis-generating rather than as a formal assessment of the certainty of evidence.

### 4.2. Future Research Directions

Despite the growing interest in parturition pain in domestic animals, substantial knowledge gaps remain. Future research should prioritise the development and validation of species-specific, multidimensional tools capable of distinguishing physiological parturition pain from pain associated with dystocia. Combining behavioural observations with facial expression analysis and with physiological and neuroendocrine indicators may provide a more comprehensive assessment of pain throughout the different stages of parturition. Finally, comparative research integrating human and veterinary medicine may further advance our understanding of the mechanisms and adaptive significance of parturition pain across mammalian species.

## 5. Conclusions

Our results show that the intensity of parturition pain remains unknown in most domestic animal species, and that a systematic comparison with the experience of women is not yet possible.

The available evidence, although limited and heterogeneous in design, is compatible with the presence of a nociceptive component during parturition in the investigated species, particularly during the expulsive phase, as suggested by the reported behavioural, physiological, and neuroendocrine changes. However, the studies specifically addressing parturition pain intensity are few, restricted to a small number of species, and often based on limited sample sizes; therefore, neither the consistency nor the magnitude of this nociceptive component can currently be established. Notably, most of these studies have been published in recent years, suggesting a growing interest in this issue. The studies in women show that behavioural assessment of parturition pain can provide reliable results, comparable to self-reported pain. This approach may provide a useful framework for the validation of parturition pain assessment methods in animals.

However, the assessment of parturition pain in animals remains extremely challenging and should initially be based, as in women, on scales originally developed for acute pathological pain, which can subsequently be adapted and validated for the specific context of parturition, with its unique physiological features. From this perspective, the definition of species-specific thresholds for physiological pain during eutocic parturition could represent a valuable tool for the early identification of pathological conditions.

Current knowledge does not allow us to suggest that physiological parturition pain should be treated routinely from an animal welfare perspective. Careful assessment of the risks and benefits of analgesia during parturition is required, since many analgesic agents may affect uterine contractility or impair neonatal viability and physiological adaptation.

## Figures and Tables

**Table 1:** Main characteristics of the retrieved studies in which parturition-related characters were measured in domestic animals.

Species	Breed	Ref.	Study Design	Country	N° of Animals	Parity	Observation Time/Stage	Aim of the Study	Investigated Parameters
Cow	Swedish Red and White, Swedish Friesian	[22]	Prospective longitudinal observational study with repeated measures	Sweden	10	primiparous	From the first signs of labour to 1 h post-partum	Association between labour phase and hormonal concentrations in heifers (*n* = 5 without obstetrical assistance vs. *n* = 5 requiring obstetrical assistance) and goats	Cortisol, catecholamines, β-endorphin, vasopressin, oxytocin
Goat	Swedish domestic				6	2 primiparous 4 multiparous			
Cow	Simmental, Simmental x Limousin	[23]	Prospective comparative observational cohort study	Germany	10	primiparous	First-stage labour	Comparison between cows and heifers during eutocic and dystocic calving (*n* = 68 cows with eutocia; *n* = 9 cows with dystocia; *n* = 10 heifers with eutocia)	Behaviour
					77	multiparous			
Cow	Holstein	[24]	Nested, frequency-matched case–control study within a prospectively monitored cohort, with repeated measurements	Canada	22	not specified	48 h before and after calving	Comparison between dystocic cows (*n* = 11) and eutocic ones (*n* = 11)	Behaviour
Cow	Double-muscled Belgian Blue	[25]	Prospective observational cohort study with repeated measures, comparing non-randomized groups	Belgium	30	multiparous	One month before the expected day of calving, and on the first, third and 14th day after calving	Comparison of pain-related indicators following natural parturition (*n* = 13) vs. caesarean section (*n* = 17)	Behaviour, vulva/flank pressure
Cow	Holstein	[26]	Retrospective comparative observational cohort study based on archived video recordings	United Kingdom	14	primiparous	Post-partum	Comparison of pain-related behaviours following assisted and unassisted labour	Behaviour
					14	multiparous			
Cow	Holstein	[27]	Prospective randomized placebo-controlled trial with repeated measurements	USA (Iowa)	20	not specified	Post-partum (within 26 h after calving)	Effect of meloxicam on post-partum pain	Gait analysis using a floor-mat-based system (MatScan, Tekscan, Inc., Boston, MA, MA)
Cow	Holstein	[28]	Prospective randomized placebo-controlled factorial study with repeated measures, including a non-randomized comparison	United Kingdom	72	not specified	Post-partum	Effect of ketoprofen on post-partum pain following assisted (*n* = 37) or unassisted (*n* = 35) deliveries	Behaviour
Goat	Swedish domestic	[29]	Prospective single-group longitudinal observational study with repeated measures	Sweden	4	multiparous	Peripartum	Association between labour phases and cardiovascular/neuroendocrine responses (*n* = 4 goats, no control group)	Blood pressure, heart rate (radiotelemetry), plasma catecholamines
Goat	Not specified	[30]	Randomized placebo-controlled trial with repeated measures and blinded behavioural assessment	Sweden	13	not specified	Parturition	Effect of buprenorphine on labour pain	Visual Analogue Scale, behaviour, vasopressin
Goat	French Alpine and Toggenburg crosses	[31]	Prospective longitudinal observational study with repeated measures	Mexico	11	primiparous	Cervical dilatation, foetal expulsion, placental expulsion	Assessment of pain-related responses in primiparous and multiparous animals	Behaviour, Grimace scale, cortisol, estradiol
					10	multiparous			
Sow	Large White, Large White crosses, Landrace crosses	[32]	Prospective comparative observational study	Finland	20	primiparous	During the 8 h following the birth of the first piglet	Comparison between savagers and non-savagers	Behaviour
Sow	Large White x Landrace	[33]	Prospective methodological observational study with repeated measures	Spain	30	primiparous	Parturition	Development of the Ease of Farrowing Score	Behaviour, body temperature
					50	multiparous			
Sow	Large White x Landrace	[34]	Prospective randomized placebo-controlled field trial with repeated measures	Spain	24	primiparous	Parturition and post-partum	Effect of meloxicam on post-partum pain	Behaviour, body temperature
					24	multiparous			
Sow	Large White × Landrace	[35]	Prospective single-cohort exploratory behavioural study with repeated within-animal measurements	United Kingdom	25	multiparous	From the birth of the first piglet until 24 h after the birth of the last piglet	Quantification of behaviours as potential pain indicators	Behaviour
Sow	Large White × Landrace	[36]	Prospective randomized assessor-blinded placebo-controlled trial with repeated measures and a non-randomized comparison	United Kingdom	24	primiparous	Peripartum	Assessment of pain and of the effect of ketoprofen during the post-partum period	Behaviour, stress biomarker
					32	multiparous			
Sow	Danbred	[37]	Prospective methodological study for scale development, with repeated within-animal observations and blinded assessment of inter- and intra-observer reliability	Spain	7	primiparous	From before the first foetal expulsion until the expulsion of the last piglet	Pain assessment based on facial expressions and body pain indicators	Facial-expression scale based on Facial Action Units (FAUs) and body pain indicators (BPIs)
					14	multiparous			
Sow	Danbred	[18]	Prospective longitudinal observational study with repeated measures and correlational analysis	Spain	5	primiparous	From before the first foetal expulsion until the expulsion of the last piglet	Pain assessment based on pain-related behaviour	Behaviour
					5	multiparous			
Horse	Polish heavy draft	[38]	Prospective single-group longitudinal observational pilot study with repeated measures	Poland	10	not specified	Post-partum following dystocic parturition	Pain of pain after fetotomy	Behaviour, physiological parameters

## Data Availability

No new data were created or analyzed in this study. Data sharing is not applicable to this article.

## Supplementary Materials

The following supporting information can be downloaded at: https://www.mdpi.com/article/10.3390/ani16162473/s1, Supplementary Material S1. PRISMA 2020 Checklist.

## Appendix A

### Appendix A.1. Animal Search Strings

#### Appendix A.1.1. Pubmed

((“Parturition”[Majr] OR “Labor, Obstetric”[Majr] OR “nesting behavior”[Mesh]

OR parturit*[tiab] OR intrapartum[tiab] OR calving[tiab] OR lambing[tiab] OR kidding[tiab] OR farrowing[tiab] OR foaling[tiab] OR whelping[tiab] OR queen-ing[tiab]

OR ((labor[tiab] OR labour[tiab])

AND (obstetric*[tiab] OR childbirth[tiab] OR parturit*[tiab] OR intrapartum[tiab])))

AND (pain[tiab] OR nocicept*[tiab] OR “Vocalization, Animal”[Mesh] OR posture[Mesh] OR pawing[tiab] OR “pain assessment”[tiab] OR “pain score”[tiab] OR “pain scores”[tiab] OR “pain scale”[tiab] OR “pain scales”[tiab] OR (ease[tiab] AND farrowing[tiab]) OR grimace[tiab] OR “grimace scale”[tiab] OR “facial expression”[tiab] OR “facial expressions”[tiab] OR analges*[tiab] OR “pain relief”[tiab]))

AND (“Animals, Domestic”[Mesh] OR “Cattle”[Mesh] OR “Sheep”[Mesh] OR “Goats”[Mesh] OR “Swine”[Mesh] OR “Horses”[Mesh] OR “Dogs”[Mesh] OR “Cats”[Mesh] OR cattle[tiab] OR bovine[tiab] OR cow[tiab] OR cows[tiab] OR heifer*[tiab] OR sheep[tiab] OR ovine[tiab] OR ewe[tiab] OR goat*[tiab] OR caprine[tiab] OR pig[tiab] OR pigs[tiab] OR sow[tiab] OR sows[tiab] OR porcine[tiab] OR swine[tiab] OR horse*[tiab] OR mare*[tiab] OR equine[tiab] OR dog[tiab] OR canine[tiab] OR bitch*[tiab] OR cat[tiab] OR feline[tiab] OR queen*[tiab]))

NOT humans[Mesh]

#### Appendix A.1.2. Scopus

(TITLE(parturit* OR parturition OR intrapartum OR childbirth OR calving OR lambing OR kidding OR farrowing OR foaling OR whelping OR queening OR labour OR

(“obstetric” W/3 (labor OR labour)) OR (“nesting” W/3 (behavior OR behaviour))) AND TITLE-ABS-KEY(pain OR nocicept* OR “pain assessment” OR “pain score” OR “pain scores” OR “pain scale” OR “pain scales” OR grimace OR “grimace scale” OR “facial expression” OR “facial expressions” OR analges* OR “pain relief” OR vocalization OR posture OR pawing OR (“farrowing ease” OR (ease W/3 farrowing)) OR (“behaviour*” W/3 scale)) AND TITLE-ABS-KEY(cattle OR bovine OR cow OR cows OR heifer* OR sheep OR ovine OR ewe OR ewes OR goat* OR caprine OR pig OR pigs OR sow OR sows OR porcine OR swine OR horse* OR mare* OR equine OR dog OR dogs OR canine OR bitch* OR cat OR cats OR feline OR queen* OR “domestic animal*” OR livestock)) AND NOT TITLE-ABS-KEY(human OR humans OR woman OR women OR patient*)

#### Appendix A.1.3. WoS

TS=((parturit* OR intrapartum OR calving OR lambing OR kidding OR farrowing OR foaling OR dystocia) NEAR/5 (pain OR nocicept* OR analges* OR “pain assessment” OR “pain scale*” OR “pain score*” OR grimace)) AND TS=(cattle OR bovine OR cow* OR heifer*

OR sheep OR ovine OR ewe*

OR goat* OR caprine

OR pig* OR sow* OR porcine OR swine

OR horse* OR mare* OR equine

OR dog* OR canine OR bitch*

OR cat* OR feline OR queen*)

NOT TS=(human OR humans OR woman OR women OR maternal OR patient*

OR meat OR carcass OR slaughter OR stunning

OR vacuum OR “meat quality”

OR debudding OR disbudding OR castration OR “tail docking”)

#### Appendix A.1.4. Embase

(‘parturition’/exp OR ‘parturition’ OR ‘labor’/exp OR ‘labor’ OR ‘nesting behavior’/exp OR ‘nesting behavior’ OR parturit*:ti,ab OR intrapartum:ti,ab OR calving:ti,ab OR lambing:ti,ab OR kidding:ti,ab OR farrowing:ti,ab OR foaling:ti,ab OR whelping:ti,ab OR queening:ti,ab OR ((labor:ti,ab OR labour:ti,ab)

AND (obstetric*:ti,ab OR childbirth:ti,ab OR parturit*:ti,ab OR intrapartum:ti,ab))) AND (pain:ti,ab OR nocicept*:ti,ab OR ‘animal vocalization’ OR ‘posture’/exp OR ‘posture’ OR pawing:ti,ab OR ‘pain assessment’:ti,ab OR ‘pain score’:ti,ab OR ‘pain scores’:ti,ab OR ‘pain scale’:ti,ab OR ‘pain scales’:ti,ab OR (ease:ti,ab AND farrowing:ti,ab) OR grimace:ti,ab OR ‘grimace scale’:ti,ab OR ‘facial expression’:ti,ab OR ‘facial expressions’:ti,ab OR analges*:ti,ab OR ‘pain relief’:ti,ab)

AND (‘domestic animal’/exp OR ‘domestic animal’ OR ‘cattle’/exp OR ‘cattle’ OR ‘sheep’/exp OR ‘sheep’ OR ‘goat’/exp OR ‘goat’ OR ‘pig’/exp OR ‘pig’ OR ‘horse’/exp OR ‘horse’ OR ‘dog’/exp OR ‘dog’ OR ‘cat’/exp OR ‘cat’ OR cattle:ti,ab OR bovine:ti,ab OR cow:ti,ab OR cows:ti,ab OR heifer*:ti,ab OR sheep:ti,ab OR ovine:ti,ab OR ewe:ti,ab OR goat*:ti,ab OR caprine:ti,ab OR pig:ti,ab OR pigs:ti,ab OR sow:ti,ab OR sows:ti,ab OR porcine:ti,ab OR swine:ti,ab OR horse*:ti,ab OR mare*:ti,ab OR equine:ti,ab OR dog:ti,ab OR canine:ti,ab OR bitch*:ti,ab OR cat:ti,ab OR feline:ti,ab OR queen*:ti,ab) NOT (‘human’/exp OR ‘human’ OR ‘human experiment’/exp OR ‘human experiment’)

## Appendix B

### Appendix B.1. Women Search Strings

#### Appendix B.1.1. Pubmed

(“Labor Pain”[Majr] OR ((“Labor, Obstetric”[Mesh] OR intrapartum[tiab] OR “first stage”[tiab] OR “second stage”[tiab] OR “active labor”[tiab] OR “active labour”[tiab])

AND (pain[tiab] OR nocicept*[tiab])))

AND ((“Pain Measurement”[Mesh] OR “Pain Threshold”[Mesh])

OR cortisol[tiab] OR “beta-endorphin”[tiab]

OR catecholamine*[tiab] OR epinephrine[tiab] OR adrenaline[tiab] OR norepinephrine[tiab] OR noradrenaline[tiab]

OR (“heart rate”[tiab] OR tachycard*[tiab])

OR (“blood pressure”[tiab] OR hypertens*[tiab])

OR (“skin conductance”[tiab] OR electrodermal[tiab])

OR pupillometr*[tiab]

OR electromyograph*[tiab] OR EMG[tiab]

OR (“observer rating”[tiab] OR observer-rated[tiab] OR “clinical assessment”[tiab] OR clinician*[tiab] OR “behavioral index”[tiab] OR behavioural[tiab] OR behavioral[tiab]))

AND (humans[Mesh] AND female[Mesh] AND pregnancy[Mesh])

AND (“pain intensity”[tiab] OR “pain severity”[tiab] OR score[tiab] OR scale[tiab])

NOT (“Self Report”[Mesh] OR self-report*[tiab] OR self-assess*[tiab] OR “patient-reported”[tiab] OR “patient reported”[tiab] OR “verbal rating”[tiab]

OR questionnaire*[tiab] OR survey*[tiab] OR interview*[tiab]

OR “Analgesia, Obstetrical”[Mesh] OR “Anesthesia, Epidural”[Mesh] OR epidural[tiab] OR analges*[tiab] OR anesthe*[tiab])

#### Appendix B.1.2. Scopus

TITLE-ABS-KEY(“labor pain” OR “labour pain”

OR ((“obstetric labor” OR “obstetric labour” OR intrapartum OR “first stage” OR “second stage” OR “active labor” OR “active labour”) AND (pain OR nocicept*)))

AND TITLE-ABS-KEY(“pain measurement” OR “pain threshold”

OR cortisol OR “beta-endorphin” OR catecholamine* OR epinephrine OR adrenaline OR norepinephrine OR noradrenaline OR “heart rate” OR tachycard* OR “blood pressure” OR hypertens* OR “skin conductance” OR electrodermal OR pupillometr* OR electromyograph* OR EMG OR “observer rating” OR “observer rated” OR observer-rated OR “clinical assessment” OR clinician* OR “behavioral index” OR behavioural OR behavioral)

AND TITLE-ABS-KEY((pregnancy OR pregnant OR gestation*)

AND (female OR woman OR women OR maternal))

AND TITLE-ABS-KEY(“pain intensity” OR “pain severity” OR score OR scale)

AND NOT TITLE-ABS-KEY(“self report” OR self-report* OR self-assess* OR

“patient-reported” OR “patient reported” OR “verbal rating” OR questionnaire* OR survey* OR interview* OR epidural OR analges* OR anesthe*)

#### Appendix B.1.3. Wos

TS=(“labor pain” OR “labour pain”

OR ((“obstetric labor” OR “obstetric labour” OR intrapartum OR “first stage” OR “second stage” OR “active labor” OR “active labour”) AND (pain OR nocicept*)))

AND TS=(“pain measurement” OR “pain threshold”

OR cortisol OR “beta-endorphin”

OR catecholamine* OR epinephrine OR adrenaline OR norepinephrine OR noradrenaline

OR “heart rate” OR tachycard*

OR “blood pressure” OR hypertens*

OR “skin conductance” OR electrodermal

OR pupillometr*

OR electromyograph* OR EMG

OR “observer rating” OR “observer rated” OR observer-rated OR “clinical assessment” OR clinician* OR “behavioral index” OR behavioural OR behavioral)

AND TS=((pregnancy OR pregnant OR gestation*)

AND (female OR woman OR women OR maternal))

AND TS=(“pain intensity” OR “pain severity” OR score OR scale)

NOT TS=(“self report” OR self-report* OR self-assess*

OR “patient-reported” OR “patient reported”

OR “verbal rating” OR questionnaire* OR survey* OR interview*

OR epidural OR analges* OR anesthe*)

#### Appendix B.1.4. Embase

(‘labor pain’/mj/exp OR ‘labor pain’ OR ((‘labor’/exp OR ‘labor’ OR intrapartum:ti,ab OR ‘first stage’:ti,ab OR ‘second stage’:ti,ab OR ‘active labor’:ti,ab OR ‘active labour’:ti,ab OR ‘obstetric labor’:ti,ab OR ‘obstetric labour’:ti,ab) AND ((labor OR labour OR childbirth OR parturition) NEAR/3 pain):ti,ab))

AND (‘pain measurement’/exp OR ‘pain measurement’ OR ‘pain threshold’/exp OR ‘pain threshold’ OR cortisol:ti,ab OR ‘beta endorphin’:ti,ab OR catecholamine*:ti,ab OR epinephrine:ti,ab OR adrenaline:ti,ab OR norepinephrine:ti,ab OR noradrenaline:ti,ab OR ‘heart rate’:ti,ab OR tachycard*:ti,ab OR ‘blood pressure’:ti,ab OR hypertens*:ti,ab OR ‘skin conductance’:ti,ab OR electrodermal:ti,ab OR pupillometr*:ti,ab OR electromyograph*:ti,ab OR EMG:ti,ab OR ‘observer rating’:ti,ab OR ‘observer rated’:ti,ab OR ‘clinical assessment’:ti,ab OR ‘pain score’:ti,ab OR ‘pain scale’:ti,ab)

AND (‘human’/exp OR ‘human’)

AND (‘female’/exp OR ‘female’)

AND (‘pregnancy’/exp OR ‘pregnancy’)

NOT (‘self report’/exp OR ‘self report’ OR ‘self report*’:ti,ab OR ‘self assess*’:ti,ab OR ‘patient-reported’:ti,ab OR ‘patient reported’:ti,ab OR ‘verbal rating’:ti,ab OR questionnaire*:ti,ab OR survey*:ti,ab OR interview*:ti,ab OR ‘obstetric analgesia’/exp OR ‘obstetric analgesia’ OR ‘epidural anesthesia’/exp OR ‘epidural anesthesia’ OR epidural:ti,ab OR analges*:ti,ab OR anesthe*:ti,ab OR apgar:ti,ab)
